# Prevalence of sufficient physical activity among general adult population and sub-populations with chronic conditions or disability in the USA

**DOI:** 10.1093/eurpub/ckad132

**Published:** 2023-08-22

**Authors:** Lijun Xu, Tianshu Li, Wenqi He, Dong Cao, Chenkai Wu, Lijie Qin

**Affiliations:** Department of Emergency, Henan Provincial People’s Hospital, People’s Hospital of Zhengzhou University, People’s Hospital of Henan University, No. 7 Weiwu Road, Jinshui District, Zhengzhou, Henan, China; Global Health Research Center, Duke Kunshan University, Jiangsu, China; Duke Global Health Institute, Durham, NC, USA; Department of Emergency, Henan Provincial People’s Hospital, People’s Hospital of Zhengzhou University, People’s Hospital of Henan University, No. 7 Weiwu Road, Jinshui District, Zhengzhou, Henan, China; Department of Emergency, Henan Provincial People’s Hospital, People’s Hospital of Zhengzhou University, People’s Hospital of Henan University, No. 7 Weiwu Road, Jinshui District, Zhengzhou, Henan, China; Global Health Research Center, Duke Kunshan University, Jiangsu, China; Department of Emergency, Henan Provincial People’s Hospital, People’s Hospital of Zhengzhou University, People’s Hospital of Henan University, No. 7 Weiwu Road, Jinshui District, Zhengzhou, Henan, China

## Abstract

**Background:**

Recently, the World Health Organization (WHO) released an updated global guideline on physical activity and sedentary behavior, including recommendations for sub-populations living with chronic conditions or disabilities. We aimed to examine the prevalence of meeting the WHO recommendations among these sub-populations in the USA.

**Methods:**

We conducted a cross-sectional study using data from the 2017 to 2018 cycle of the National Health and Nutrition Examination Survey (NHANES).

**Results:**

We revealed variations in physical activity levels among individuals with chronic conditions and disability. US adults with diabetes, hypertension or disability had a lower prevalence of recommended physical activity levels than the general population. In addition, certain demographic groups such as being female, older and having lower socioeconomic status were associated with a lower likelihood of meeting the WHO recommendations on physical activity.

**Conclusions:**

Our findings underscore the importance of promoting physical activity levels among US adults, especially those with older age, low socioeconomic status, hypertension and disability.

## Introduction

Low physical activity has been a leading risk factor for non-communicable diseases.^1^ A sufficient amount of physical activity may reduce the risk of mortality, disability and chronic conditions.^2^^,^^3^ Globally, one in four adults does not meet the recommendations for physical activity.^2^^,^^3^ In the USA, nearly 20% of adults were physically inactive, ranging from 17.3% in Colorado to 47.7% in Puerto Rico.^2^ To address this health risk at its source, a global target regarding the decreased levels of physical inactivity is adopted in Global Action Plan on Physical Activity 2018–2030.^4^ In November 2020, the World Health Organization (WHO) released the updated global guidelines on physical activity and sedentary behavior to facilitate the new target of a 15% relative reduction in physical inactivity by 2030.^5^ For the first time, this new guideline includes recommendations for sub-populations, such as people living with chronic conditions (e.g. diabetes, hypertension, cancer) or disability.^6^ This guideline provides a reference for evaluating physical activity levels in vulnerable sub-populations.

The present study, using a nationally representative sample, aims to examine the prevalence of meeting physical activity recommendations among US adults, including those living with chronic conditions (i.e. diabetes, hypertension, cancer) or disability, respectively, according to the WHO 2020 guidelines on physical activity and sedentary behavior. We also investigated the socio-demographic and health correlates of compliance with the new physical activity guideline among US adults.

## Methods

### Data source and study participants

Data were from the 2017 to 2018 cycle of the National Health and Nutrition Examination Survey (NHANES), which includes a series of cross-sectional studies with multistage probability sampling to achieve a nationally representative sample of non-institutionalized residents in the USA.^7^ All participants aged ≥18 years who participated in the physical activity survey were eligible. Of 5856 eligible participants, we further excluded 14 persons who answered ‘Don’t know’ to either of the three questions in the physical activity questionnaire regarding whether they did vigorous-intensity sports, moderate-intensity sports and walked or cycled for at least 10 min continuously in a typical week. The final analytic sample consisted of 5842 participants.

### Physical activity

According to the WHO 2020 guidelines on physical activity and sedentary behavior, all adults (aged ≥18 years) should undertake at least 150–300 min of moderate-intensity exercise or 75–150 min of vigorous-intensity activity or an equivalent combination of moderate-intensity and vigorous-intensity aerobic physical activity in a typical week to meet the recommendation.^6^ This recommendation also applies to adults with hypertension, diabetes, cancer or disability.^6^ We classified physical activity levels in a typical week into three categories: insufficient, sufficient and highly sufficient (table 1). The exercise time was coded as 0 if participants answered ‘Don’t know’ to questions regarding the exact number of days or minutes they spent on specific physical activity in a typical week.

**Table 1:** Definition of physical activity level for US adults (similarly applied to those with cancer, diabetes, hypertension and disability)

	Physical activity levels		
	Insufficiently	Sufficiently	Highly
Vigorous-intensity activity, min/week^a^	<75	75–150	>150
Moderate-intensity activity, min/week^b^	<150	150–300	>300
Equivalent combination, min/week^c^	<150	150–300	>300

a Vigorous-intensity activity = no. of days of vigorous recreational activities × no. of minutes of vigorous recreational activities per day.

b Moderate-intensity activity = no. of days of moderate recreational activities × no. of minutes of vigorous recreational activities per day + no. of days of walk or bicycle × no. of minutes of walk or bicycle per day.

c Equivalent combination = 2 × no. of time of vigorous-intensity activity + no. of time of moderate-intensity activity, and this formula was used when neither the time of vigorous-intensity physical activity nor the time of moderate-intensity physical activity met the guideline.

### Diabetes

The NHANES comprises interviews, laboratory tests and physical examinations administered by highly trained staff. Participants had their blood drawn in the morning in the mobile examination center. Glycohemoglobin (HbA1c) was measured in a fasting subsample of participants 12 years or above. Whole blood specimens were processed, stored and shipped to the University of Missouri-Columbia, MO, for analysis. Diabetes was defined as HbA1c ≥6.5%, physician diagnosis of diabetes (self-report), or current use of oral hypoglycemic medications or insulin (self-report).

### Hypertension

Trained examiners took three blood pressure (BP) measurements in a seated position using a mercury sphygmomanometer with an appropriate cuff size in the mobile examination center. If only one BP reading was recorded, the reading was considered as the mean value; if more than one BP reading was obtained, the average of all measurements was taken. Hypertension was defined as a systolic BP of ≥130 mmHg, diastolic BP of ≥80 mmHg, physician diagnosis of hypertension (self-report), or current use of antihypertensive medication (self-report).

### Cancer

Participants were considering having cancer if they responded ‘Yes’ to the question, ‘Have you ever been told by a doctor or other health professional that you had cancer or a malignancy of any kind?’ Skin cancer was excluded.

### Disability

According to the guideline, disability includes three dimensions: impairment, activity limitation and participation restrictions.^6^ Due to extensive missing data contained in the Physical Functioning dataset, we only used data from the Disability dataset to define disability. Participants were considered having a disability if they answered ‘Yes’ to one of the following questions: whether they have severe difficulty hearing, seeing even when wearing glasses, concentrating/remembering/making decisions, walking/climbing stairs, dressing/bathing or doing errands alone.

### Socio-demographic characteristics

Socio-demographic characteristics were collected using computer-assisted interviews at home. Sex (male or female), age (18–44, 45–64 or ≥65 years), race/ethnicity (Hispanic, non-Hispanic White, non-Hispanic Black or other race), family income poverty ratio (<1.30, 1.30 to <1.85, 1.85 to <3.00 or ≥3.00), education (less than high school, high school or equivalent or more than high school), marital status (married or living with partner, widowed, divorced or separated or never married) were self-reported.

### Statistical analysis

We estimated the prevalence of US adults with insufficient, sufficient and highly sufficient physical activity. We also calculated the prevalence of each of the three physical activity levels by socio-demographics, including sex, age, race/ethnicity, family income poverty ratio, education and marital status. We used the chi-square test to examine the association of each socio-demographic characteristic and physical activity level (insufficient, sufficient and highly sufficient). We repeated these analyses for adults with diabetes, hypertension, cancer and disability. Using the total sample, we conducted the logistic regression model to analyze the association of socio-demographics and chronic conditions (diabetes, hypertension, cancer and disability) with the physical activity level (sufficient or highly sufficient vs. insufficient physical activity) using the total sample.

We calculated prevalence estimates as weighted proportions; 95% confidence intervals were computed with variance estimated using the Taylor series linearization method following NHANES recommended procedures and weights.^7^^,^^8^ Sampling weight, strata and primary sampling unit parameters were appropriately specified to account for the complex, multistage, probability sampling design of the NHANES to obtain US nationally representative estimates for the general population and sub-populations. Stata/SE 16.0 and RStudio version 1.4.1103 were used for all statistical analyses in January 2021.

## Results

### Prevalence of physical activity levels in the general US population

Among 5842 US adults, 55.1%, 18.9% and 26.1% had insufficient, sufficient and highly sufficient physical activity levels, respectively (table 2). Male participants who were younger, socio-economically advantaged (higher education level and higher family income poverty ratio) and never married were more likely to have higher level of physical activity. The prevalence of meeting recommendations for physical activity (sufficient or highly sufficient) was 40.3% for females and 49.9% for males. Over half of adults aged 18–44 years met the physical activity recommendations (53.0%) and 36.1% had highly sufficient physical activity. More than half of the US adults with a family income poverty ratio ≥3.00 had a sufficient or highly sufficient physical activity level in a week (50.2%). Less than one-third of US adults without a high school diploma engaged in a recommended amount of physical activity in a week (29.5%), but the majority of those having a more than high school education reached the recommended level (52.0%).

**Table 2:** Prevalence of insufficient, sufficient, highly sufficient physical activity by demographics, NHANES 2017–18

		Weighted prevalence (95% CI)			
	Subgroup (%)	Insufficiently physical activity	Sufficiently physical activity	Highly physical activity	Recommended physical activity
Total		55.1 (52.2–57.9)	18.9 (17.0–20.8)	26.1 (24.4–27.9)	44.9 (42.1–47.8)
**Sex** ***					
Male	2835 (48.2)	50.1 (46.0–54.2)	18.8 (16.6–20.9)	31.1 (28.1–34.2)	49.9 (45.8–54.0)
Female	3007 (51.8)	59.7 (56.5–62.9)	18.9 (16.7–21.1)	21.4 (19.0–23.8)	40.3 (37.1–43.5)
**Age, years** ***					
18–44	2377 (46.1)	47.0 (43.1–50.8)	16.9 (14.9–18.9)	36.1 (33.3–39.0)	53.0 (49.2–56.9)
45–64	1969 (34.3)	59.8 (54.7–65.0)	21.8 (17.4–26.3)	18.3 (15.8–20.9)	40.2 (35.0–45.3)
≥65	1496 (19.6)	65.7 (61.7–69.8)	18.2 (14.8–21.6)	6.1 (11.6–20.5)	34.2 (30.2–38.3)
**Race/ethnicity**					
Hispanic	1332 (16.1)	55.3 (50.1–60.5)	17.1 (14.5–19.7)	27.6 (23.6–31.6)	44.7 (39.5–49.9)
Non-Hispanic White	2028 (62.1)	55.3 (51.7–58.9)	19.4 (16.6–22.3)	25.3 (23.0–27.6)	44.7 (41.1–48.3)
Non-Hispanic Black	1338 (11.3)	55.0 (51.5–58.5)	17.1 (14.8–19.5)	27.9 (24.9–30.8)	45.0 (41.5–48.5)
Other races	1144 (10.5)	53.5 (46.8–60.2)	19.9 (14.9–24.8)	26.7 (22.8–30.5)	46.5 (39.8–53.2)
**Family income poverty ratio** **					
<1.30	1461 (20.8)	60.4 (52.9–68.0)	14.2 (11.3–17.1)	25.4 (18.4–32.4)	39.6 (32.0–47.1)
1.30 to <1.85	767 (10.5)	62.2 (57.8–66.5)	15.7 (12.1–19.3)	22.1 (18.1–26.0)	37.8 (33.5–42.2)
1.85 to <3.00	982 (18.0)	58.9 (55.0–62.8)	18.2 (15.4–21.1)	22.9 (19.5–26.3)	41.1 (37.2–45.0)
≥3.00	1800 (50.8)	49.8 (46.3–53.3)	22.0 (18.1–25.9)	28.2 (26.0–30.4)	50.2 (46.7–53.7)
**Education level** ***					
Less than high school	1182 (11.7)	70.5 (65.0–76.0)	11.3 (8.0–14.5)	18.3 (14.6–21.9)	29.5 (24.0–35.0)
High school or equivalent	1465 (28.0)	63.7 (61.5–65.8)	13.3 (11.3–15.3)	23.0 (19.8–26.3)	36.3 (34.2–38.5)
More than high school	3182 (60.3)	48.0 (44.4–51.6)	22.9 (20.3–25.6)	29.1 (26.6–31.5)	52.0 (48.4–55.6)
**Marital status** ***					
Married	3245 (62.0)	56.7 (54.2–59.1)	20.7 (17.9–23.6)	22.6 (20.4–24.7)	43.3 (40.9–45.8)
Widowed	462 (5.9)	75.0 (68.0–81.9)	13.3 (8.1–18.5)	11.8 (5.9–17.6)	25.0 (18.1–32.0)
Divorced or separated	838 (13.0)	58.2 (51.7–64.7)	17.2 (12.2–22.2)	24.6 (19.7–29.6)	41.8 (35.3–48.3)
Never married	1005 (19.2)	44.7 (38.3–51.2)	16.9 (12.7–21.0)	38.4 (33.7–43.1)	55.3 (48.8–61.7)

CI, confidence interval.

*** *P* < 0.001,

** *P* < 0.01,

* *P* < 0.05 for the comparison in physical activity level (insufficiently, sufficiently, highly) within each variable.

### Prevalence of physical activity levels in sub-populations with chronic conditions or disability

**Figure 1:**
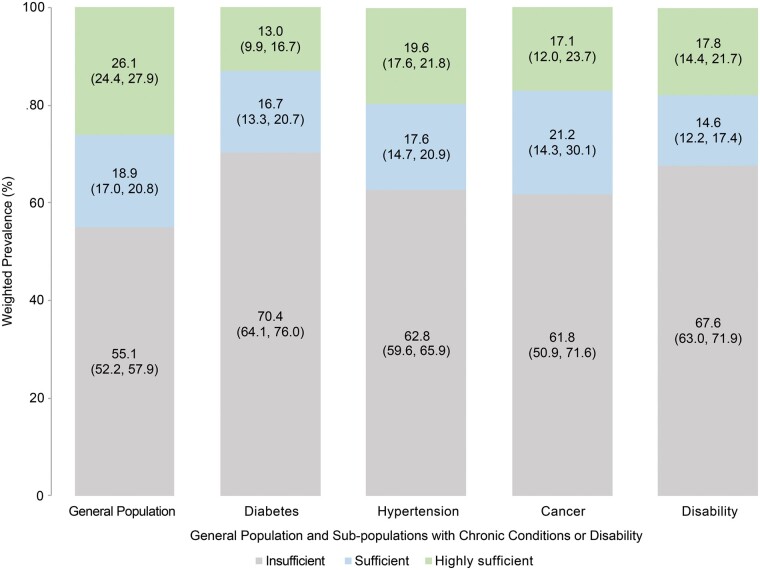
Prevalence of insufficient, sufficient and highly sufficient physical activity level in general population and sub-populations with chronic conditions or disability. *Note*: Statistically significant difference of prevalence was reached in sub-population with diabetes (*P* = 0.001), sub-population with hypertension (*P* < 0.001), sub-population with disability (*P* = 0.001), but not in sub-population with cancer (*P* = 0.08), compared with general population

US adults with hypertension, diabetes or disability had significantly lower prevalence of recommended physical activity level (sufficient or highly sufficient) than the general population. We did not find a statistically significant difference in the prevalence of recommended physical activity levels between persons with cancer and the general population. The prevalence of recommended physical activity levels was 29.6%, 37.2%, 38.2% and 32.4% among diabetes, hypertension, cancer and disability, respectively (figure 1).

Among adults with diabetes, those who were male, more educated and never married were more likely to have higher physical activity levels (Supplementary table S1). Among adults with hypertension, those who were younger, male, socio-economically advantaged (higher education level and family income poverty ratio) and never married were more likely to have higher level of physical activity (Supplementary table S2). The proportion of adults with cancer reaching the recommendations for physical activity was the highest among the four disease groups. Among adults with cancer, those who were socio-economically advantaged (higher education level and family income poverty ratio) had a higher physical activity level (Supplementary table S3). Among adults with disability, those who were male, younger, and more educated were more likely to have higher physical activity levels (Supplementary table S4).

### Socio-demographic predictors in sufficient or highly sufficient physical activity levels

In the multivariable-adjusted logistic regression model, females, older age, lower family income ratio, and lower education level, hypertension and disability were significantly associated with lower odds of meeting the recommendations for physical activity (sufficient or highly sufficient) (table 3). Females had 31% lower odds of having sufficient or highly sufficient physical activity levels than males. US adults aged 45–64 years had 28% lower odds of meeting the recommended physical activity level than those aged 18–44 years. Those with a family income poverty ratio <1.30 had 39% lower odds of meeting the recommendations on physical activity than those with a poverty ratio ≥3.00. Adults without high school education had 104% lower odds of meeting the recommended physical activity level than those with an education level above high school. Adults with hypertension and diabetes had 34% and 35% lower odds of meeting the physical activity recommendations than the general population.

**Table 3:** Results of the logistic regression analyses examining socio-demographic predictors in sufficient or highly sufficient physical activity levels (NHANES 2017–18)

	Sufficiently or highly physical activity vs. insufficiently physical activity	
	OR (95% CI)	*P* value
**Sex**		
Female vs. male	0.69 (0.53–0.89)	0.007**
**Age, years**		
18–44	Ref	
45–64	0.72 (0.56–0.91)	0.009**
≥65	0.79 (0.56–1.10)	0.147
**Race/ethnicity**		
Hispanic	Ref	
Non-Hispanic White	0.85 (0.66–1.08)	0.170
Non-Hispanic Black	1.00 (0.70–1.43)	0.990
Other race	0.83 (0.55–1.25)	0.340
**Family income poverty ratio**		
<1.30	Ref	
1.30 to <1.85	1.02 (0.72–1.45)	0.891
1.85 to <3.00	1.11 (0.87–1.41)	0.370
≥3.00	1.39 (1.10–1.75)	0.008**
**Education level**		
Less than high school	Ref	
High school or equivalent	1.20 (0.90–1.60)	0.201
More than high school	2.04 (1.50–2.76)	<0.001***
**Marital status**		
Married or living with partner	Ref	
Widowed	0.92 (0.60–1.43)	0.703
Divorced or separated	1.24 (0.90–1.72)	0.167
Never married	1.19 (0.88–1.61)	0.232
**Health condition**		
Hypertension	0.66 (0.48–0.89)	0.011*
Disability	0.65 (0.45–0.94)	0.026*
Diabetes	0.78 (0.59–1.04)	0.084
Cancer	0.90 (0.56–1.45)	0.644

*** *P* < 0.001,

** *P* < 0.01,

* *P* < 0.05.

## Discussion

The present study aimed to evaluate the compliance with the new WHO 2020 guidelines on physical activity and sedentary behavior among US adults and individuals living with chronic conditions (hypertension, diabetes and cancer) or disability. We found that 44.9% of US adults met the recommended physical activity levels, and the prevalence was 29.6%, 37.2%, 38.2% and 32.4% among those with diabetes, hypertension, cancer and disability, respectively. We revealed variations in physical activity levels by chronic conditions and disability; adults with hypertension and disability had low compliance with physical activity at recommended levels. Besides, we identified several socio-demographic sub-groups with a low prevalence of sufficient physical activity levels.

We found that levels of physical activity in adults with hypertension were lower than in the general population, which was in line with a previous study investigating the influence of hypertension on physical activity in the USA.^9^ This cross-sectional study involving 391 017 adults showed a lower prevalence of sufficient or highly sufficient physical activity among hypertensive individuals, but the estimate (60.2%) was much higher than ours (37.2%).^9^ This discrepancy was partially because we excluded domestic duties in our study. Moreover, we witnessed a low compliance with the new guidelines among hypertensive individuals. Reasons for low levels of physical activity among sub-population with hypertension may include lack of interest in exercise, lack of enjoyment from physical activities, fear of injury or pain and perceived barriers (including exercise milieu, time expenditure, physical exertion and family discouragement).^10–12^ Interventions focusing on improving the physical activity level among hypertensive individuals are needed.

Our study showed that the prevalence of the recommended level of physical activity is lower among disabled adults compared to the non-disabled. These results were consistent with several previous studies.^13–15^ Reasons for lower levels of physical activity might include (i) personal barriers such as pain, lack of energy and the perception that exercise is too difficult; (ii) environmental barriers such as discriminatory practices at fitness centers and other recreational venues.^16^^,^^17^ These barriers may prevent disabled adults from engaging in physical activity sufficiently. Considering the beneficial effects of physical activity, such as the improvement of stamina and muscle strength for sub-populations with a disability, we recommend promoting an inclusive approach to overcome these barriers and support their needs for physical activity.

We found variations in levels of physical activity by gender and age in both the general population and in sub-populations with diabetes, hypertension or disability. Women had a lower prevalence of meeting recommendations on physical activity. This finding was echoed by Caspersen et al., who found that levels of physical inactivity for women were 5.5% greater than for men.^18^ The low compliance may result from social barriers such as the lack of self-efficacy and social support that impact women more in the USA.^19^^,^^20^ Moreover, older adults were less likely to meet recommendations for physical activity. This finding is consistent with previous studies that age was associated with decreased physical activity,^20^ which is probably driven by physiological, psychosocial and environmental barriers throughout the lifespan.^21–23^ Variations in physical activity by socio-demographics suggest that women and older people are high-risk groups and emphasize the need to increase physical activity through targeted interventions considering these barriers.

We found that the prevalence of sufficient or highly sufficient physical activity levels varied by education level and family income. Adults who were more educated had a higher likelihood of sufficient or highly sufficient physical activity levels, which is in line with existing literature.^24–26^ Moreover, we found that family income was positively related to the prevalence of having recommended physical activity levels. This finding was echoed by a previous study showing that adults in the high-income stratum (annual income ≥$75 000) were 1.9 times more likely to meet the recommendations on physical activity than their lower-income counterparts.^27^ Both findings provided additional evidence to highlight the disparities in health behaviors by socioeconomic status.^28^

We acknowledge several limitations. First, physical activity was self-reported in the NHANES, which is subjective to recall bias. However, several previous studies have demonstrated the reliability and validity of self-reported physical activity.^29^^,^^30^ Second, we only considered aerobic exercise due to data unavailability. Future studies may include muscle-strengthening activities to provide a complete picture of physical activity levels. Third, we did not include mental disorders in the sub-population living with disability due to the large amount of missing data in the 2017–18 cycle of NHANES. Future investigations may classify adults with mental disorders into the disability group.

## Conclusion

The present study was among the first to examine the prevalence of sufficient physical activity among US adults along with sub-populations living with chronic conditions—diabetes, hypertension and cancer—and disability according to the new WHO 2020 guidelines on physical activity and sedentary behavior. We found that physical activity levels varied by chronic conditions and disability. For example, at recommended levels, adults with hypertension and disability had lower compliance with physical activity. In addition, US adults who were female, older and less socio-economically advantaged could be less likely to meet the recommendations on physical activity. These findings highlight the importance of promoting physical activity levels among US adults, especially those with older age, low socioeconomic status, hypertension and disability.

## Supplementary Material

ckad132_Supplementary_Data

## Data Availability

The datasets used in this study can be found in the 2017–18 National Health and Nutrition Examination Survey (NHANES) at the official website: https://wwwn.cdc.gov/nchs/nhanes/continuousnhanes/default.aspx?BeginYear=2017.
