# Supplementary material for: Prevalence of sufficient physical activity among general adult population and sub-populations with chronic conditions or disability in the USA
Source: Eur J Public Health. 2023 Aug 22;33(5):891–6. doi: 10.1093/eurpub/ckad132 (PMC10567242; doi:10.1093/eurpub/ckad132)
Supplement: ckad132_Supplementary_Data [file ckad132_supplementary_data.docx]

**S1 Table. Prevalence of Insufficient, Sufficient, Highly Sufficient Physical Activity by Demographics in Sub-populations with Diabetes, NHANES 2017-2018.**

|  |  | **Prevalence, % (95%CI)** | | | |
| --- | --- | --- | --- | --- | --- |
|  | **Subgroup (%)** | **Insufficiently**  **Physical Activity** | **Sufficiently**  **Physical Activity** | **Highly**  **Physical Activity** | **Recommended**  **Physical Activity** |
| **Total** | 1,106 (15.1) | 70.4 (64.1, 76.0) | 16.7 (13.3, 20.7) | 13.0 (9.9, 16.7) | 29.6 (24.0, 35.9) |
| **Sex**** |  |  |  |  |  |
| Male | 569 (50.4) | 62.4 (56.0, 68.7) | 21.4 (16.5, 26.2) | 16.3 (11.8, 20.7) | 37.6 (31.3, 44.0) |
| Female | 537 (49.6) | 78.5 (71.0, 86.0) | 11.9 (6.4, 17.4) | 9.6 (5.4, 13.8) | 21.5 (14.0, 29.0) |
| **Age, years** |  |  |  |  |  |
| 18-44 | 112 (14.3) | 70.1 (55.2, 84.9) | 13.2 (4.6, 21.8) | 16.7 (5.2, 28.2) | 29.9 (15.1, 44.8) |
| 45-64 | 469 (43.9) | 64.4 (55.2, 73.5) | 22.1 (14.9, 29.3) | 13.5 (9.0, 18.0) | 35.6 (26.5, 44.8) |
| ≥65 | 525 (41.8) | 76.8 (70.9, 82.7) | 12.1 (7.7, 16.6) | 11.1 (5.0, 17.1) | 23.2 (17.3, 29.1) |
| **Race/ethnicity** |  |  |  |  |  |
| Hispanic | 269 (15.4) | 67.6 (59.6, 75.6) | 16.9 (11.0, 22.7) | 15.6 (8.5, 22.6) | 32.4 (24.4, 40.4) |
| Non-Hispanic White | 349 (59.5) | 72.5 (62.8, 82.2) | 16.7 (10.7, 22.7) | 10.8 (5.6, 15.9) | 27.5 (17.8, 37.2) |
| Non-Hispanic Black | 269 (13.0) | 69.9 (63.4, 76.3) | 14.5 (9.5, 19.5) | 15.6 (10.0, 21.3) | 30.1 (23.7, 36.6) |
| Other races | 219 (12.0) | 64.0 (53.8, 74.1) | 18.4 (7.1, 29.7) | 17.7 (9.2, 26.1) | 36.0 (25.9, 46.2) |
| **Family income poverty ratio** |  |  |  |  |  |
| <1.30  1.30 to <1.85  1.85 to <3.00  ≥3.00 | 267 (19.6)  166 (12.4)  198 (21.6)  321 (46.4) | 75.1 (65.4, 84.7)  81.3 (73.3, 89.4)  64.2 (51.9, 76.5)  70.2 (60.6, 79.9) | 12.9 (7.7, 18.2)  9.8 (3.9, 15.8)  21.5 (9.6, 33.4)  15.5 (9.0, 22.0) | 12.0 (3.8, 20.3)  8.8 (4.0, 13.7)  14.3 (6.8, 21.9)  14.3 (8.3, 20.3) | 24.9 (15.3, 34.6)  18.7 (10.6, 26.7)  35.8 (23.5, 48.1)  29.8 (20.1, 39.4) |
| **Education level*** |  |  |  |  |  |
| Less than high school | 298 (17.0) | 82.2 (75.3, 89.0) | 7.3 (4.0, 10.6) | 10.6 (5.8, 15.4) | 17.8 (11.0, 24.7) |
| High school or equivalent | 257 (29.5) | 74.0 (64.6, 83.3) | 14.4 (7.1, 21.7) | 11.6 (5.4, 17.9) | 26.0 (16.7, 35.4) |
| More than high school | 548 (53.6) | 64.8 (57.4, 72.3) | 20.9 (16.4, 25.4) | 14.3 (8.5, 20.0) | 35.2 (27.7, 42.6) |
| **Marital status**** |  |  |  |  |  |
| Married or living with partner | 670 (66.9) | 68.6 (60.6, 76.6) | 19.6 (14.6, 24.5) | 11.9 (8.0, 15.7) | 31.4 (23.4, 39.4) |
| Widowed | 143 (11.5) | 88.4 (81.5, 95.3) | 8.2 (2.3, 14.2) | 3.3 (0, 7.4) | 11.6 (4.7, 18.5) |
| Divorced or separated | 190 (14.0) | 67.5 (57,4, 77.6) | 12.3 (0.7, 23.8) | 20.2 (11.3, 29.1) | 32.5 (22.4, 42.6) |
| Never married | 98 (7.5) | 63.3 (48.3, 78.3) | 12.3 (4.6, 20.0) | 24.4 (11.5, 37.3) | 36.7 (21.7, 51.7) |

****p*<.001, ***p*<.01, **p*<.05 for the comparison in physical activity level (insufficiently, sufficiently, highly) within each variable.

**S2 Table. Prevalence of Insufficient, Sufficient, Highly Sufficient Physical Activity by Demographics in Sub-populations with Hypertension, NHANES 2017-2018.**

|  |  | **Prevalence, % (95%CI)** | | | |
| --- | --- | --- | --- | --- | --- |
|  | **Subgroup (%)** | **Insufficiently**  **Physical Activity** | **Sufficiently**  **Physical Activity** | **Highly**  **Physical Activity** | **Recommended**  **Physical Activity** |
| **Total** | 3,203 (52.6) | 62.8 (59.6, 65.9) | 17.6 (14.7, 20.9) | 19.6 (17.6, 21.8) | 37.2 (34.1, 40.4) |
| **Sex**** |  |  |  |  |  |
| Male | 1,666 (53.0) | 57.0 (52.8, 61.3) | 18.8 (14.7, 23.0) | 24.1 (21.1, 27.1) | 43.0 (38.7, 47.2) |
| Female | 1,537 (47.0) | 69.3 (64.8, 73.8) | 16.2 (13.7, 18.8) | 14.5 (11.4, 17.6) | 30.7 (26.2, 35.2) |
| **Age, years**** |  |  |  |  |  |
| 18-44 | 668 (26.6) | 55.9 (50.5, 61.3) | 15.2 (10.5, 19.9) | 28.9 (25.8, 31.9) | 44.1 (38.7, 49.5) |
| 45-64 | 1,311 (42.9) | 63.2 (58.4, 68.1) | 20.6 (16.3, 24.9) | 16.2 (13.1, 19.3) | 36.8 (31.9, 41.6) |
| ≥65 | 1,224 (30.5) | 68.1 (62.5, 73.7) | 15.6 (12.0, 19.1) | 16.3 (11.3, 21.3) | 31.9 (26.3, 37.5) |
| **Race/ethnicity** |  |  |  |  |  |
| Hispanic | 622 (13.1) | 62.3 (54.2, 70.4) | 18.0 (12.1, 23.8) | 19.8 (15.4, 24.2) | 37.7 (29.6, 45.8) |
| Non-Hispanic White | 1,141 (63.3) | 63.1 (58.6, 67.6) | 17.8 (13.3, 22.3) | 19.1 (16.0, 22.3) | 36.9 (32.4, 41.4) |
| Non-Hispanic Black | 873 (13.3) | 62.5 (58.3, 66.7) | 16.3 (12.4, 20.1) | 21.2 (18.3, 24.1) | 37.5 (33.3, 41.7) |
| Other races | 567 (10.3) | 61.9 (55.7, 68.2) | 17.8 (12.5, 23.1) | 20.2 (16.4, 24.1) | 38.1 (31.8, 44.3) |
| **Family income poverty ratio**** |  |  |  |  |  |
| <1.30  1.30 to <1.85  1.85 to <3.00  ≥3.00 | 771 (19.7)  424 (10.1)  578 (19.6)  993 (50.6) | 70.6 (62.8, 78.5)  70.2 (62.9, 77.5)  64.8 (58.3, 71.3)  57.8 (54.0, 61.5) | 13.0 (7.8, 18.2)  10.6 (7.1, 14.2)  17.1 (11.0, 23.3)  21.1 (17.0, 25.1) | 16.4 (10.5, 22.3)  19.2 (12.4, 25.9)  18.1 (14.2, 21.9)  21.1 (18.6, 23.7) | 29.4 (21.5, 37.2)  29.8 (22.5, 37.1)  35.2 (28.7, 41.7)  42.2 (38.5, 46.0) |
| **Education level**** |  |  |  |  |  |
| Less than high school | 668 (12.1) | 74.8 (66.7, 82.9) | 11.5 (5.9, 17.2) | 13.7 (9.4. 17.9) | 25.2 (17.1, 33.3) |
| High school or equivalent | 806 (29.3) | 71.0 (67.0, 75.1) | 13.2 (8.4, 18.0) | 15.8 (11.8, 19.8) | 29.0 (24.9, 33.0) |
| More than high school | 1,721 (58.6) | 56.2 (53.1, 59.2) | 21.1 (17.5, 24.7) | 22.7 (19.4, 26.0) | 43.8 (40.8, 46.9) |
| **Marital status*** |  |  |  |  |  |
| Married or living with partner | 1,838 (63.7) | 62.2 (58.4, 66.0) | 19.5 (14.9, 24.1) | 18.3 (16.0, 20.5) | 37.8 (34.0, 41.6) |
| Widowed | 376 (9.3) | 73.3 (63.6, 83.0) | 13.7 (7.3, 20.0) | 13.0 (5.4, 20.7) | 26.7 (17.0, 36.4) |
| Divorced or separated | 558 (14.8) | 61.7 (55.6, 67.8) | 16.8 (10.5, 23.0) | 21.5 (15.2, 27.9) | 38.3 (32.2, 44.4) |
| Never married | 402 (12.1) | 59.7 (52.3, 67.2) | 12.2 (8.3,16.1) | 28.1 (21.6, 34.5) | 40.3 (32.8, 47.7) |

****p*<.001, ***p*<.01, **p*<.05 for the comparison in physical activity level (insufficiently, sufficiently, highly) within each variable.

**S3 Table. Prevalence of Insufficient, Sufficient, Highly Sufficient Physical Activity by Demographics in Sub-populations with Cancer, NHANES 2017-2018.**

|  |  | **Prevalence, % (95%CI)** | | | |
| --- | --- | --- | --- | --- | --- |
|  | **Subgroup (%)** | **Insufficiently**  **Physical Activity** | **Sufficiently**  **Physical Activity** | **Highly**  **Physical Activity** | **Recommended**  **Physical Activity** |
| **Total** | 473 (8.4) | 61.8 (50.9, 71.6) | 21.2 (14.3, 30.1) | 17.1 (12.0, 23.7) | 38.2 (28.4, 49.1) |
| **Sex** |  |  |  |  |  |
| Male | 219 (40.0) | 52.3 (42.0, 62.6) | 26.0 (13.4, 38.6) | 21.7 (11.8, 31.6) | 47.7 (37.4, 58.0) |
| Female | 254 (60.0) | 68.1 (55.6, 80.6) | 17.9 (8.7, 27.1) | 14.0 (6.6, 21.4) | 31.9 (19.4, 44.4) |
| **Age, years** |  |  |  |  |  |
| 18-44 | 41 (12.0) | 43.5 (16.4, 70.6) | 27.7 (2.2, 53.3) | 28.8 (11.5, 46.1) | 56.5 (29.4, 83.6) |
| 45-64 | 147 (36.3) | 54.1 (33.8, 74.3) | 25.6 (10.6, 40.5) | 20.4 (8.6, 32.2) | 45.9 (25.7, 66.2) |
| ≥65 | 285 (51.7) | 71.4 (62.7, 80.2) | 16.5 (9.7, 23.4) | 12.0 (5.2, 18.8) | 28.6 (19.8, 37.3) |
| **Race/ethnicity** |  |  |  |  |  |
| Hispanic | 73 (8.0) | 63.0 (48.4, 77.6) | 21.3 (4.5, 38.2) | 15.6 (5.3, 26.0) | 37.0 (22.4, 51.6) |
| Non-Hispanic White | 251 (78.4) | 61.2 (49.8, 72.6) | 22.4 (14.1, 30.6) | 16.4 (9.1, 23.8) | 38.8 (27.4, 50.2) |
| Non-Hispanic Black | 94 (7.4) | 60.5 (47.9, 73.0) | 17.4 (7.1, 27.7) | 22.2 (12.0, 32.3) | 39.5 (27.0, 52.1) |
| Other races | 55 (6.2) | 69.0 (51.8, 86.1) | 10.2 (3.8, 16.6) | 20.9 (6.8, 34.9) | 31.0 (13.9, 48.2) |
| **Family income poverty ratio*** |  |  |  |  |  |
| <1.30  1.30 to <1.85  1.85 to <3.00  ≥3.00 | 103 (15.2)  60 (8.1)  88 (15.0)  168 (61.8) | 67.7 (56.5, 78.8)  88.5 (81.9, 95.2)  62.5 (52.5, 72.5)  62.1 (45.5, 78.7) | 7.2 (0.4, 14.1)  5.7 (0, 11.7)  21.2 (8.6, 33.9)  26.0 (12.2, 39.8) | 25.1 (10.7, 39.5)  5.7 (0.9, 10.5)  16.2 (7.8, 24.7)  11.9 (5.1, 18.7) | 32.3 (21.2, 43.5)  11.5 (4.8, 18.1)  37.5 (27.5, 47.5)  37.9 (21.3, 54.5) |
| **Education level*** |  |  |  |  |  |
| Less than high school | 75 (8.1) | 76.1 (57.8, 94.5) | 14.7 (0, 31.4) | 9.1 (1.6, 16.6) | 23.9 (5.5, 42.2) |
| High school or equivalent | 118 (27.2) | 78.9 (64.4, 93.4) | 8.0 (3.1, 12.8) | 13.2 (1.9, 24.4) | 21.1 (6.6, 35.6) |
| More than high school | 279 (64.7) | 52.8 (41.2, 64.3) | 27.5 (18.5, 36.5) | 19.7 (11.0, 28.4) | 47.2 (35.7, 58.8) |
| **Marital status** |  |  |  |  |  |
| Married or living with partner | 260 (60.0) | 59.4 (45.2, 73.6) | 23.5 (11.7, 35.4) | 17.1 (10.6, 23.5) | 40.6 (26.4, 54.8) |
| Widowed | 82 (14.5) | 80.7 (72.5, 88.9) | 9.9 (1.7, 18.1) | 9.4 (1.0, 17.7) | 19.3 (11.1, 27.5) |
| Divorced or separated | 92 (18.8) | 54.9 (32.1, 77.6) | 19.6 (1.4, 37.8) | 25.5 (10.8, 40.3) | 45.1 (22.4, 67.9) |
| Never married | 38 (6.7) | 61.0 (25.5, 96.5) | 28.7 (0, 68.4) | 10.3 (0, 23.1) | 39.0 (3.5, 74.5) |

^a^ Skin cancer was excluded.

****p*<.001, ***p*<.01, **p*<.05 for the comparison in physical activity level (insufficiently, sufficiently, highly) within each variable.

**S4 Table. Prevalence of Insufficient, Sufficient, Highly Sufficient Physical Activity by Demographics in Sub-populations with Disability, NHANES 2017-2018.**

|  |  | **Prevalence, % (95%CI)** | | | |
| --- | --- | --- | --- | --- | --- |
|  | **Subgroup (%)** | **Insufficiently**  **Physical Activity** | **Sufficiently**  **Physical Activity** | **Highly**  **Physical Activity** | **Recommended**  **Physical Activity** |
| **Total** | 1,798 (8.4) | 67.6 (63.0, 71.9) | 14.6 (12.2, 17.4) | 17.8 (14.4, 21.7) | 32.4 (28.1, 37.0) |
| **Sex**** |  |  |  |  |  |
| Male | 861 (45.0) | 62.3 (56.1, 68.5) | 15.2 (11.4, 19.0) | 22.5 (16.5, 28.5) | 37.7 (31.5, 43.9) |
| Female | 937 (55.0) | 72.0 (66.8, 77.1) | 14.1 (11.0, 17.3) | 13.9 (10.7, 17.1) | 28.0 (22.9, 33.2) |
| **Age, years***** |  |  |  |  |  |
| 18-44 | 410 (30.8) | 56.0 (50.0, 62.1) | 13.2 (8.7, 17.7) | 30.8 (23.9, 37.7) | 44.0 (37.9, 50.1) |
| 45-64 | 616 (32.1) | 71.7 (64.0, 79.4) | 15.2 (9.8, 20.6) | 13.1 (7.3, 18.9) | 28.3 (20.6, 36.0) |
| ≥65 | 772 (37.1) | 73.8 (68.3, 79.3) | 15.2 (10.6, 19.8) | 11.0 (6.8, 15.2) | 26.2 (20.7, 31.7) |
| **Race/ethnicity** |  |  |  |  |  |
| Hispanic | 409 (15.2) | 68.2 (64.1, 72.4) | 13.1 (9.1, 17.2) | 18.6 (13.6, 23.6) | 31.8 (27.6, 35.9) |
| Non-Hispanic White | 769 (64.6) | 67.0 (61.6, 72.5) | 15.3 (11.0, 19.6) | 17.7 (13.8, 21.5) | 33.0 (27.5, 38.4) |
| Non-Hispanic Black | 379 (10.7) | 69.3 (62.4, 76.2) | 15.7 (10.9, 20.5) | 15.1 (9.6, 20.5) | 30.7 (23.8, 37.6) |
| Other races | 241 (9.5) | 68.9 (55.5, 82.3) | 11.0 (5.2, 16.7) | 20.2 (9.8, 30.6) | 31.1 (17.7, 44.5) |
| **Family income poverty ratio** |  |  |  |  |  |
| <1.30  1.30 to <1.85  1.85 to <3.00  ≥3.00 | 598 (31.8)  279 (13.2)  300 (19.9)  364 (35.1) | 70.3 (64.1, 76.5)  73.8 (65.3, 82.3)  55.4 (57.7, 73.2)  62.0 (54.2, 69.8) | 11.1 (7.5, 14.6)  11.7 (7.6, 15.9)  20.5 (12.8, 28.3)  17.1 (11.2, 22.9) | 18.6 (12.3, 24.9)  14.5 (6.2, 22.7)  24.0 (9.2, 18.8)  20.9 (15.0, 26.8) | 29.7 (23.5, 35.9)  26.2 (17.7, 34.7)  34.6 (26.8, 42.3)  38.0 (30.2, 45.8) |
| **Education level*** |  |  |  |  |  |
| Less than high school | 529 (18.9) | 78.9 (70.9, 86.9) | 8.1 (5.3, 10.9) | 13.0 (7.4, 18.5) | 21.1 (13.1, 29.1) |
| High school or equivalent | 493 (34.1) | 69.7 (64.1, 75.3) | 12.7 (8.4, 17.0) | 17.6 (12.5, 22.7) | 30.3 (24.7, 35.9) |
| More than high school | 767 (47.0) | 61.5 (53.5, 69.6) | 18.7 (14.5, 22.9) | 19.8 (14.0, 25.6) | 38.5 (30.4, 46.5) |
| **Marital status** |  |  |  |  |  |
| Married or living with partner | 876 (54.3) | 67.7 (62.2, 73.1) | 14.8 (10.5, 19.1) | 17.5 (12.8, 22.2) | 32.3 (26.9, 37.8) |
| Widowed | 257 (12.6) | 80.0 (71.4, 88.5) | 12.9 (6.9, 18.9) | 7.1 (0.6, 13.6) | 20.0 (11.5, 28.6) |
| Divorced or separated | 350 (17.2) | 64.4 (55.1, 73.6) | 16.3 (9.5, 23.2) | 19.3 (11.6, 27.0) | 35.6 (26.4, 44.9) |
| Never married | 258 (15.9) | 63.3 (55.4, 71.2) | 14.3 (6.8, 21.8) | 22.4 (14.9, 30.0) | 36.7 (28.8, 44.6) |

****p*<.001, ***p*<.01, **p*<.05 for the comparison in physical activity level (insufficiently, sufficiently, highly) within each variable.
